# β-Hemolysin/Cytolysin of Group B *Streptococcus* Enhances Host Inflammation but Is Dispensable for Establishment of Urinary Tract Infection

**DOI:** 10.1371/journal.pone.0059091

**Published:** 2013-03-07

**Authors:** Ritwij Kulkarni, Tara M. Randis, Swati Antala, Alice Wang, Fábio E. Amaral, Adam J. Ratner

**Affiliations:** Department of Pediatrics, Columbia University, New York, New York, United States of America; Louisiana State University, United States of America

## Abstract

Group B Streptococcus (GBS; *Streptococcus agalactiae*) is a major human pathogen that disproportionately affects neonates and women in the peripartum period and is an emerging cause of infection in older adults. The primary toxin of GBS, β-hemolysin/cytolysin (βH/C), has a well-defined role in the pathogenesis of invasive disease, but its role in urinary tract infection (UTI) is unknown. Using both in vitro and in vivo models, we analyzed the importance of βH/C in GBS uropathogenesis. There were no significant differences in bacterial density from the bladders or kidneys from mice infected with wild-type or isogenic βH/C-deficient GBS, and competitive indices from co-infection experiments were near 1. Thus, βH/C is dispensable for the establishment of GBS-UTI. However, βH/C-sufficient GBS induced a more robust proinflammatory cytokine response in cultured bladder epithelial cells and in the urinary tracts of infected mice. Given the near ubiquity of βH/C-expressing strains in epidemiologic studies and the importance of local inflammation in dictating outcomes and sequelae of UTI, we hypothesize that βH/C-driven inflammatory signaling may be important in the clinical course of GBS-UTI.

## Introduction

*Streptococcus agalactiae* (Group B *Streptococcus*, GBS) is a human pathogen with particular importance in the perinatal and neonatal period. GBS is the leading cause of invasive diseases including pneumonia, meningitis and sepsis among newborns and of peripartum and postpartum infections among women [1]. In addition, there is a growing appreciation of the role of GBS as a pathogen of non-pregnant adults, particularly among the elderly and those with diabetes mellitus [2]. Due to the establishment of guidelines for screening and intrapartum antibiotic prophylaxis in pregnancy, the incidence of early-onset GBS diseases of neonates has decreased, but the burden of adult GBS diseases has remained substantial [3]. GBS is a cause of urinary tract infections (UTI) in adults, and clinical presentations may range from asymptomatic bacteriuria to cystitis, pyelonephritis, and urosepsis. In pregnant women, UTI caused by GBS is associated with high-level vaginorectal colonization and is a risk factor for complications including chorioamnionitis and neonatal GBS disease [4], [5]. Despite its clinical importance, a detailed understanding of the pathogenesis of GBS-UTI remains elusive. In several studies employing murine models, GBS has been demonstrated to bind urothelium and activate a bladder cytokine response within 24 h of infection [6]–[8]. Such responses appear to be distinct from and relatively modest compared to those elicited by uropathogenic *Escherichia coli* [8]. Klein et al. found that capsular sialic acid was a critical component of GBS-mediated inflammation during infection [7], but the role of other virulence factors remains unclear.

In this study, we undertook a comprehensive analysis of the role of the pore-forming β-hemolysin/cytolysin (βH/C) in GBS UTI. Nearly all clinical GBS isolates produce βH/C, which is an important virulence factor involved in the invasion of human epithelial cells and release of pro-inflammatory cytokines [9]. βH/C is involved in many clinical sequelae associated with GBS infection such as meningitis and sepsis [10]. Consistent with prior reports [7], we noted that βH/C was not required for establishment of UTI in a murine model. However, we found that GBS induces proinflammatory cytokine production in a βH/C-dependent manner both in vitro and in vivo. Because of its effect on host inflammatory responses, GBS βH/C may be an important factor in UTI pathogenesis and sequelae.

## Methods

### Ethics Statement

This study was approved by the Columbia University Institutional Animal Care and Use Committee.

### Bacterial strains, cell lines and reagents

GBS wild type (WT) strain CNCTC 10/84 (1169-NT1; ATCC 49447, serotype V) [11], the isogenic, βH/C-deficient *cylE*Δ*cat* mutant (Δ*cylE*), and the trans-complemented strain Δ*cylE* +pJC10.E [12] were generous gifts from Dr. Victor Nizet (University of California, San Diego). We generated a spontaneous streptomycin mutant of the WT strain for use in animal infection experiments. In order to prepare the frozen starter cultures, overnight cultures of GBS were diluted 1∶40 in sterile tryptic soy (TS) broth, grown to OD_600_ = 0.6, and stored in 1 ml aliquots in TS with 20% sterile glycerol at −80°C. On the day of the experiment, the frozen starters of GBS strains were thawed, washed in TS broth, and grown in fresh TS broth at 37°C without shaking to OD_600_ = 0.6–0.8. Bacterial density was confirmed by serial dilution with quantitative culture. Human bladder epithelial carcinoma cell line 5637 was purchased from ATCC (HTB-9) and maintained in RPMI (ATCC) with 10% fetal bovine serum at 37°C in a humidified atmosphere with 5% CO_2_.

### Mouse model of ascending UTI

6–8 week old female C57Bl/6J mice were infected with 10^7^ colony-forming units (CFU) of GBS WT or Δ*cylE* in 50 μl volume instilled at a slow rate (10 µl/sec) by transurethral catheterization using a well-lubricated sterile soft polyethylene catheter. Mice were euthanized 1, or 5 days post-infection, and bladders, kidneys and urine samples were collected in a sterile fashion. Serial dilutions of organ homogenates or urine samples were cultured on TS agar plates in order to quantify bacterial load. Bladder or kidney halves were stored in RNAlater (Ambion) for RNA isolation using the RNAqueous-4-PCR (Ambion) kit according to the manufacturer's instructions. For competition experiments, WT and KO bacteria were mixed in a 1∶1 ratio prior to introduction into transurethral infection. At 1, or 5 days post-infection organs (bladder and kidneys) were harvested from each infected mouse. Each organ homogenate was plated on TS agar to determine total GBS density and on TS agar supplemented with 100 µg/ml streptomycin to determine specifically the density of the WT strain. Competitive index (CI) was calculated as [(WT CFU recovered/WT CFU inoculated) ÷ (Δ*cylE* CFU recovered/ Δ*cylE* CFU inoculated)]. A CI value of 1 indicates equivalent fitness; CI>1 indicates a WT fitness advantage, and CI<1 indicates a Δ*cylE* fitness advantage.

### Epithelial cell adherence and cytokine transcription assays

Confluent ATCC 5637 cell monolayers in 24 well polystyrene plates were washed with serum-free RPMI medium. Cells were then infected with log-phase cultures of WT or Δ*cylE* strains (MOI  = 10) and incubated at 37°C, 5% CO_2_ for 1 h. Bacterial inocula were enumerated by dilution plating on TS agar plates (Inoculum CFU; IC). Following the incubation, cell-free supernatants were collected for lactate dehydrogenase (LDH) assay to determine cell death (Roche). 5637 cells were washed three times to remove non-adherent bacteria and adherent CFUs determined (Adherent CFU; AC). % adhesion was determined as (AC÷IC) ×100. RNA from washed 5637 cells was extracted as described below.

### RNA extraction, reverse transcription and quantitative real-time PCR (qRTPCR)

Total RNA from mouse organs or 5637 human bladder carcinoma cell line was extracted using the RNAqueous-4-PCR RNA extraction kit as above. RNA was reverse transcribed to cDNA using the high capacity cDNA reverse transcription kit (Applied Biosystems.) qRT-PCR was carried out using Power SYBR-Green Master Mix in a StepOne Plus thermal cycler (Applied Biosystems). The list of primers used for qRT-PCR is shown in the Table 1. Relative quantification (RQ) values were calculated using a comparative threshold cycle (*ΔΔCT*) program on the StepOne software version 2.0.

**Table 1:** Primers used for qRT-PCR.

Primer Name*	Sequence	Reference
hIL-1α FOR	GAC TCA GGC TTA AGC TGC CA	this study
hIL-1α REV	CCT TCC CGT TGG TTG CTA CT	
hIL-6 FOR	AAG AGT AAC ATG TGT GAA AGC	[18]
hIL-6 REV	CTA CTC TCA AAT CTG TTC TGG	
hIL-8 FOR	TTG GCA GCC TTC CTG ATT TC	[18]
hIL-8 REV	TAT GCA CTG ACA TCT AAG TTC TTT AG	
hGAPDH FOR	GGGCGC CTG GTC ACC AGG GCT G	[18]
hGAPDH REV	GGG GCC ATC CAC AGT CTT CTG	
mIFNb FOR	AAC TCC ACC AGC AGA CAG TG	[18]
mIFNb REV	GTG GAG AGC AGT TGA GGA CA	
mRANTES FOR	TCG TGC CCA CGT CAA GGAGTA TTT	this study
mRANTES REV	TCT TCT CTG GGT TGG CAC ACA CTT	
mKC FOR	CCG CGC CTA TCG CCA ATG AGC TGC GC	this study
mKC REV	CTT GGG GAC ACC TTA GCA TCT TTT GG	
mMCP-1 FOR	ATC CCA ATG AGT AGG CTG GAG AGC	this study
mMCP-1 REV	CAG AAG TGC TTG AGG TGG TTG TG	
mIL-6 FOR	GGT CCT GGT CCT TAG CCA CTC	[18]
mIL-6 REV	TGA TGC ACT TGC AGA AAA CAA	
mTNF-α FOR	ATG AGC ACA GAA AGC ATG ATC	this study
mTNF-α REV	TAC AGG CTT GTC ACT CGA ATT	
mIL-1α FOR	CGA AGA CTA CAG TTC TGC CAT T	this study
mIL-1α REV	GAC GTT TCA GCG GTT CTC AGA G	
mActin FOR	CCT TTG AAA AGA AAT TTG TCC	[18]
mActin REV	AGA AAC CAG AAC TGA AAC TGG	

* m and h denote mouse and human specificity of the primers.

### Statistical Analysis

CFU/ml data from animal infections were compared using Mann-Whitney test, cytokine transcription and adherence frequency data were compared by t test or ANOVA using Prism 4 software (GraphPad). A *P*-value ≤0.05 was considered significant.

## Results

### βH/C-expressing GBS induce epithelial cell cytokine production

**Figure 1:**
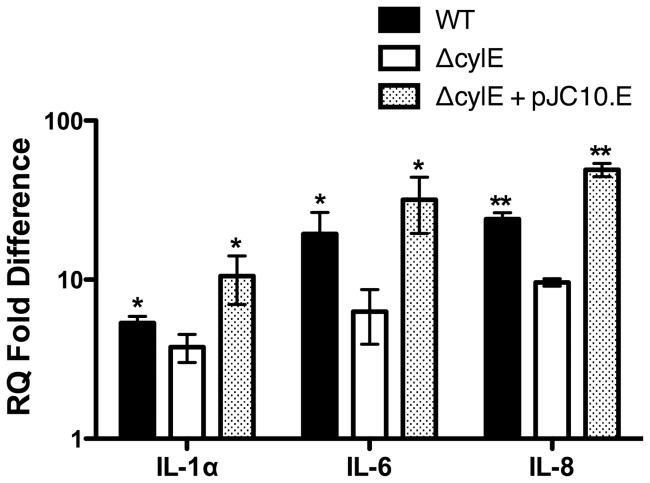
GBS βH/C is important for induction of cytokine mRNA in human bladder epithelial cells. Monolayers of 5637 cells were exposed to medium alone, WT, Δ*cylE*, or complemented GBS strains for 1 h. Transcript levels for different proinflammatory cytokines were determined using qRT-PCR with normalization to GAPDH. RQ values were calculated by comparative CT (ΔΔCT), and fold difference over transcript levels in bacteria exposed to medium alone was determined. RQ fold difference values from triplicate readings in a representative experiment (from three biological replicates) ± standard deviation are shown. WT and complemented strains were compared to Δ*cylE* by two-tailed t tests. (*P* values for this and subsequent figures are denoted as follows: *, *P*≤0.05, **, *P*≤0.01, while NS refers to P>0.05).

**Figure 2:**
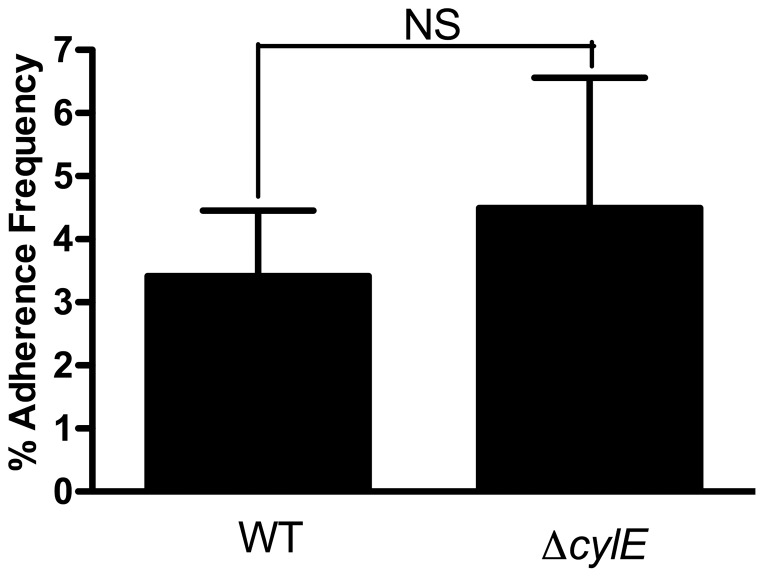
GBS adhere to epithelial cells in a βH/C-independent fashion. Following exposure of 5637 cells to WT or Δ*cylE* GBS for 1 h, the monolayers were washed and the number of bacterial adherent to the cells was determined by quantitative culture plating. Adherence frequency was determined as the ratio of adherent to inoculum CFU, expressed as a percentage.

Using a human bladder carcinoma cell line, we observed that WT GBS induced significantly more transcription of interleukin (IL)-6, IL-8, and, to a lesser extent, IL-1α, than medium alone or the Δ*cylE* deletion mutant (Fig. 1). Importantly, the proinflammatory activity was restored to WT-like levels upon infection with *trans*-complemented Δ*cylE* strain (Fig. 1). At this time point (1 h) there were no significant differences in cytotoxicity induced by WT, Δ*cylE* deletion mutant, or the complemented strain as determined by LDH release assay, indicating that even at sublethal concentrations, βH/C may promote inflammation. WT bacteria caused discernably more lysis in bladder cells than Δ*cylE* only after 4 h of incubation (data not shown). We also noted that following 1 h incubation, equivalent numbers (CFU/ml) of WT and Δ*cylE* bacteria had adhered to 5637 human bladder cell line (Fig. 2). These data indicate that βH/C is not involved in the adherence of GBS to bladder epithelial cells but may modulate host cell signaling.

### βH/C is dispensable for GBS UTI

**Figure 3:**
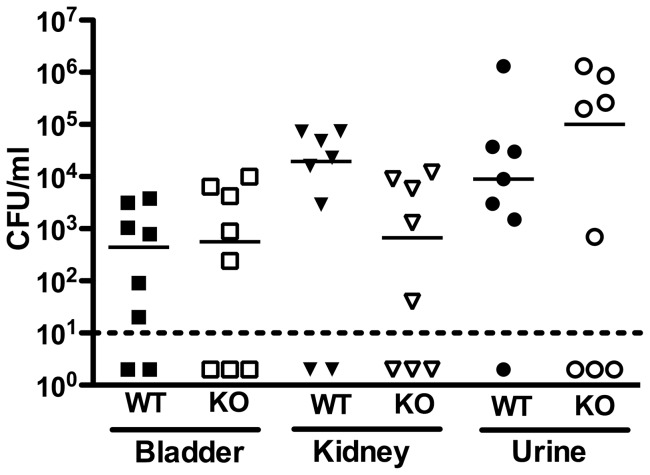
GBS βH/C is dispensable for establishment of UTI in a murine model. 6–8 week old female C57/Bl6J mice were infected with WT or Δ*cylE* GBS strains separately by transurethral catheterization and colonization assessed at 24 h. No difference was observed in the number of CFUs recovered from bladders, kidneys or urine comparing WT (filled shapes) with Δ*cylE* (open shapes) GBS.

The proinflammatory nature of βH/C argues in favor of a role for this cytotoxin as an important virulence factor with the relevance to infection in the urinary tract. We hypothesized that in comparison to its WT counterpart, Δ*cylE* GBS strain would be at a disadvantage in a mouse model of ascending urinary tract infection. However, when separately infected with 10^7^ CFU/ml of WT or Δ*cylE* strains for 24 h, we observe no significant differences by Mann-Whitney U test in number of bacteria recovered from the bladder (*P* = 0.72), kidney (*P* = 0.10), or urine (*P* = 0.96) of the animals (Fig. 3), indicating that production of βH/C is not required for establishment or maintenance of UTI.

**Figure 4:**
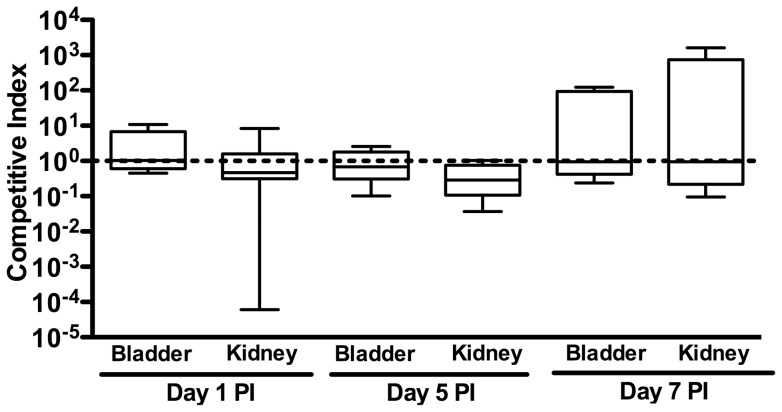
No fitness advantage of βH/C-sufficient GBS during UTI at early or late time points. Competition assays were performed using WT and Δ*cylE* GBS and revealed competitive indices near 1, indicating an absence of fitness differences, at 1 and 5 days post-infection.

In order to assess whether βH/C confers a subtle fitness advantage during UTI, we performed in vivo competition assays. On days 1 and 5 following infection, we found that CI values remain tightly clustered around 1, indicating that neither WT nor Δ*cylE* has a fitness advantage in the mouse model of ascending UTI (Fig. 4).

**Figure 5:**
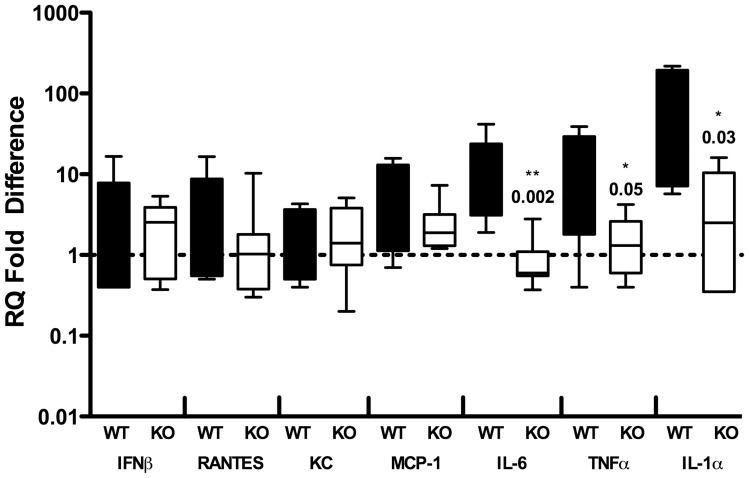
Enhanced bladder inflammatory responses to WT compared to βH/C-deficient GBS. Bladder tissue from WT-infected (filled boxes) animals was assessed by qRT-PCR at 24 h and demonstrated increased transcription of IL-6, IL-1α and TNF-α compared to those receiving Δ*cylE* GBS (empty boxes). Data were compared using Mann-Whitney *U* test; *P* values are indicated atop the boxes where appropriate.

Bacterial density and host response are both drivers of pathology during UTI in vivo, so we measured transcription of cytokine mRNA from murine bladders during single infection studies. Consistent with our in vitro observations using a bladder cell line, we noted significant transcriptional upregulation of proinflammatory cytokines such as IL-6, TNF-α and IL-1α (Fig. 5) in the setting of WT GBS infection, exceeding that induced by Δ*cylE*, even in the setting of similar bacterial density. This finding implicates βH/C as a potential driver of host inflammation in the urinary tract.

## Discussion

The kidneys, bladder, and urine are sterile under normal conditions, but infection of these sites is exceedingly common and may be associated with substantial morbidity, especially in vulnerable patient populations. Understanding the pathogenesis of UTI is important to guide future preventive and therapeutic strategies, and a wealth of information has become available in recent years regarding both bacterial strategies for survival [13] and host detection of and defense against infection [14]. Most such studies have focused on *E. coli* and other enteric Gram-negative rods, as these are the most common agents of UTI in humans. However, it is also important to delineate the pathogenesis of less frequently isolated uropathogens, especially those such as GBS, which may affect disproportionately populations such as pregnant women and the elderly.

Clinically important GBS produce βH/C, a pore-forming cytotoxin that is involved in the induction of apoptosis, cytokine production, and neutrophil recruitment and is crucial for virulence in a number of diseases including pneumonia, sepsis, and meningitis [10]. We hypothesized that this factor would also have a role in the establishment and maintenance of GBS UTI. However, findings from our in vitro and in vivo assays indicated that βH/C is dispensable for epithelial cell adherence, bladder colonization, and ascending infection of the kidney. These findings were consistent with data described by Klein et al. [7]. In our experiments, competition assays designed to reveal subtle fitness defects supported these conclusions as well.

Induction of local cytokine responses is a crucial first response to bacterial infections, and epithelial signaling in the bladder recruits professional immune cells required for efficient clearance of infection [15]. We found that even in the setting of equivalent bacterial numbers, the βH/C-deficient mutant was severely attenuated in induction of inflammation in both of our models. Inflammation drives UTI-associated symptoms [16], [17], and chronic infection and sequelae distant to the urinary tract (such as premature labor) are correlated with GBS UTI and may be dependent on the magnitude or duration of inflammatory responses [17]. Thus, even in the absence of a direct effect on bacterial density during infection, βH/C may play an important role in UTI pathogenesis and is deserving of further investigation.
